# ST-segment elevation myocardial infarction heart of Charlotte one-year (STEMI HOC-1) study: a prospective study protocol

**DOI:** 10.1186/s12872-023-03416-3

**Published:** 2023-08-11

**Authors:** Marheb Badianyama, Arthur Mutyaba, Samantha Nel, Nqoba Tsabedze

**Affiliations:** Division of Cardiology, Department of Internal Medicine, School of Clinical Medicine, Faculty of Health Sciences, University of the Witwatersrand, 2193 Johannesburg, South Africa

**Keywords:** ST-segment elevation myocardial infarction, Acute coronary syndrome, Mortality, Sub-Saharan Africa, Biomarkers, Thrombolysis, Percutaneous coronary intervention

## Abstract

**Background:**

ST-segment elevation myocardial infarction (STEMI) is a clinically distinguishable yet lethal sequela of ischaemic heart disease (IHD). In sub-Saharan Africa (SSA), death due to acute STEMI is increasing. In South Africa, there is a paucity of data available on the clinical outcomes of acute STEMI within one year for individuals treated in the public healthcare sector. This study primarily seeks to determine the one-year all-cause mortality rate of acute STEMI. The study also assesses the value of serum cardiac biomarkers of myocardial damage and serum uric acid in predicting all-cause mortality in STEMI.

**Methods:**

This is a single-centre observational prospective cohort of all consecutive individuals presenting with an acute STEMI to the Charlotte Maxeke Johannesburg Academic Hospital (CMJAH) in Johannesburg, South Africa. Research data will be sourced on admission through electronic medical records, blood laboratory results and coronary angiography reports, and at follow-up through periodic telephonic interviews and standardised echocardiograms. At least 355 eligible participants will be continuously followed over one year, and clinical outcomes will be measured 30 days, three months, six months and one year after the index hospitalisation.

**Discussion:**

This study provides insights into the demographic, risk factors and clinical profiles of individuals with STEMI in South Africa. Its findings may improve the risk stratification, prognostication, and therapeutic management of STEMI patients in our setting. By comparing the clinical outcomes between the different coronary reperfusion strategies, our results may guide clinicians in providing better patient treatment, particularly in sub-Saharan Africa, where access to percutaneous coronary intervention may be limited. Furthermore, the study offers insights into the routine use of baseline serum uric acid as a potential low-cost prognostic biomarker of all-cause mortality in STEMI. Finally, this study’s findings may be of public health significance to local policymakers to aid in reinforcing primary prevention strategies and developing structured referral networks for timely coronary reperfusion of acute STEMI.

**Supplementary Information:**

The online version contains supplementary material available at 10.1186/s12872-023-03416-3.

## Background

There has been considerable developments in the last 15 years in the treatment of ST-segment elevation myocardial infarction (STEMI), more particularly the timelines to reperfusion therapy, the application of emergent primary percutaneous coronary intervention (PPCI), the widespread shift away from streptokinase use to tissue plasminogen activators, the implementation of a pharmaco-invasive strategy, advances in antiplatelet therapy and cardiac rehabilitation [1].

An acute STEMI is a fatal and clinically distinguishable sequela of ischaemic heart disease (IHD). In the absence of contraindications, the treatment of choice for a STEMI is prompt reperfusion by percutaneous coronary intervention (PCI) or timeous thrombolytic therapy [2].

Unlike in high-income countries (HIC), where STEMI-related deaths are declining, in sub-Saharan Africa (SSA), STEMI mortality is on the rise [3, 4]. Moreover, in SSA, death due to cardiovascular diseases has superseded death due to communicable diseases [5]. Although the in-hospital mortality rate of STEMI has been reported in SSA, data on outpatient follow-up in this population is primarily confined to HIC. This study aims to determine the clinical outcomes in STEMI patients within a year of treatment at the Charlotte Maxeke Johannesburg Academic Hospital (CMJAH).

This research is significant due to the paucity of contemporary data on the one-year clinical outcomes of STEMI patients treated in South Africa. Notably, the consequential prevalence of HIV infection and tuberculosis in our setting will provide a unique insight into the relationship between these disease processes, the biomarkers of myocardial damage and the clinical outcomes within a year of an acute STEMI presentation .

Data from the South African Registry of Acute Coronary Events multinational Survey of current management Strategies (ACCESS) reported that the thirty-day and one-year outpatient all-cause mortality rates in STEMI patients were 2.4% and 6.7%, respectively [6]. The French Registry on Acute ST-elevation Myocardial Infarction (FAST-MI) of 2008 reported that in patients treated with thrombolysis, the thirty-day mortality rate is 9.2% without PCI and 3.9% when rescue PCI is subsequently performed (p = 0.044) compared to 2.8% in those who undergo a pharmaco-invasive approach (p = 0.147) [7]. According to the FAST-MI of 2008, the crude one-year survival rate is 78.5% in patients without reperfusion, 93.6% for thrombolysis, and 91.8% for PPCI (p < 0.001 for overall comparison; p = 0.31 for thrombolysis vs PPCI). When compared to other regions in SSA, the crude one-year mortality rate of 6.7% reported in the South African ACCESS registry was lower than the overall death rate of 10.4% reported in a single national referral centre study conducted in the Intensive Care Unit of Abidjan Heart Institute, in Côte d’ Ivoire [8].

An age of ≥ 70 years, female gender and heart failure on admission are significant factors predictive of mortality at thirty days and one-year follow-up of STEMI, irrespective of myocardial revascularisation strategy [8]. Diabetes mellitus on admission and a history of stroke or transient ischaemic attack (TIA) are also associated with a higher risk of death at one year [6]. In SSA, delayed access to emergency medical services (EMS) and healthcare facilities with reperfusion therapy limits favourable outcomes in STEMI patients [9]. In a local prospective cohort study at a single tertiary centre involving 100 STEMI patients, Meel et al. reported that only 37% of eligible patients received fibrinolytic therapy [10]. Only 3% received the medication within one hour. The median total time delay to administering a fibrinolytic agent was 270 min (45–584), with patient and transport delays being the most crucial reasons. For fibrinolytic therapy not given within one hour of hospital arrival, the calculated number of lives lost due to these delays was 32 patients per 1000 treated.

The Thrombolysis in Myocardial Infarction (TIMI) risk score for STEMI is a bedside risk assessment tool that predicts 30-day crude mortality [11]. This score is based on eight clinical variables obtained from the clinical history, physical examination, electrocardiogram (ECG) and laboratory testing. The score ranges from 0 to 14. The 30-day mortality is 0.8% with a score of zero and 35.9% with scores greater than eight (p < 0.001).

The Global Registry of Acute Coronary Events (GRACE) risk score for STEMI predicts the six-month risk of crude mortality. This score involves eight variables from the clinical history, physical examnation, ECG and laboratory testing, and it stratifies patients into three risk categories (low risk: < 109 points, moderate risk: 109–140 points, and high risk: > 140 points). The recently adopted GRACE risk score 2.0 predicts the risk of death one year or three years after a STEMI but substitutes the Killip class and serum creatinine with diuretic usage and a history of renal dysfunction, respectively. The median predicted one-year mortality risks of low, moderate and high-risk groups are 1.6%, 2.8% and 5.9%, respectively [12].

In addition, discontinuation of evidence-based medication (such as aspirin, beta-blockers, statins and angiotensin-converting enzyme inhibitors) one month beyond hospitalisation is associated with higher twelve-month mortality (hazards ratio, 3.81; 95% CI 1.88–7.72; p < 0.001) [13].

Serum cardiac biochemical markers (biomarkers) of myocardial damage and atherosclerosis, including high-sensitivity cardiac troponin T and I (hs-cTnT and hs-cTnI), C - reactive protein (CRP), and serum uric acid (SUA), have proven to be helpful for prognostication in STEMI patients.

Admission serum hs-cTnT or hs-cTnI is strongly predictive of death, non-fatal AMI and revascularisation at 30 days (hazard ratio 5.16, 95% CI 2.25–11.9, p < 0.001) and one year (hazard ratio 2.88, 95% CI 1.79–4.63, p < 0.001), even after adjusting for baseline risk factors such as age, sex, race and time from symptom onset to index hs-cTnT or I measurement [14].

Treatment guidelines recommend measuring serum CRP to aid in diagnosing and treating STEMI patients. STEMI patients with serum CRP > 3.0 mg/dL at or after discharge have a significantly higher risk of re-infarction, ischaemic stroke, coronary artery occlusion and death within two years of the index AMI [15]. The risk of mortality increases with higher CRP values at admission (Odds Ratio 1.04, 95% CI 0.91–1.20, p < 0.0001) and 30 days (Odds Ratio 1.12, 95% CI 0.93–1.34, p = 0.007) after hospital discharge [16].

In a systematic review and meta-analysis involving nine studies, Trkulja et al. showed that ‘high’ baseline SUA independently predicts all-cause mortality, re-infarction, angina, revascularisation and cardiac arrest at 30 days (Odds Ratio 2.26, 95% CI 1.85–2.77, p < 0.001), and at both 6 and 12 months (Hazard Ratio 1.30, 95% CI 1.01–1.68, p = 0.042) [17]. ‘High’ SUA refers to values in the hyperurecaemic, i.e., > 0.42 mmol/L and > 0.36 mmol/L or high-normal range, i.e., 0.31 mmol/L-0.42 mmol/L and 0.25 mmol/L -0.36 mmol/L in men and women, respectively. However, this study provided no data on the association between serial measurements of SUA at 30 days, six months and one year after hospital discharge and clinical outcomes in STEMI patients. Moreover, the combination of these three serum cardiac biomarkers might have an incremental value in the risk stratification of these patients.

Primary percutaneous coronary intervention (PPCI) is emergent PCI without prior fibrinolysis. It is the current reperfusion strategy of choice in STEMI patients who present within 12 h of symptom onset, provided it is conducted expeditiously within 120 min from the time of STEMI diagnosis [4]. A study by Zhu et al. showed that PPCI significantly reduces mortality, non-fatal re-infarction, and stroke by approximately 50% at 6 to 12 months (Odds Ratio 0.52, 95% CI 0.44–0.62, p < 0.001) [18].

However, fibrinolysis remains an important alternative reperfusion strategy that prevents early mortality in 30 per 1000 patients treated within 6 h after symptom onset. Fibrinolytic therapy should be administered within 12 h of symptom onset, provided that early primary PCI cannot be performed and there are no contraindications [19].

Facilitated PCI refers to routine early PCI < 12 h after reperfusion with either fibrinolysis or glycoprotein IIb/IIIa (GPIIb/IIIa) receptor inhibitors, regardless of the pharmacologic reperfusion outcome [20]. The Strategic Reperfusion Early After Myocardial Infarction (STREAM) study has shown that adopting a pharmaco-invasive strategy (i.e., fibrinolysis followed by coronary angiography or possible rescue PCI ≥ 2 h after failed fibrinolysis) offers similar benefits in all-cause thirty-day mortality when compared with primary PCI [21]. A pharmaco-invasive approach is safer and has less risk of 30-day mortality, non-fatal re-infarction, stroke, and major bleeding than facilitated PCI and fibrinolytic therapy alone (Odds Ratio for death 0.79, 95% CI 0.59–1.08; p < 0.14 when compared to fibrinolysis) [20]. Revascularisation using either thrombolysis or a pharmaco- invasive approach is common in low-to-middle-income countries (LMIC).

The Minimising Adverse Haemorrhagic Events by Transradial Access site and systemic Implementation of AngioX: Radial vs. Femoral (MATRIX: Radial vs. Femoral) randomised controlled trial has provided robust evidence in morbidity and mortality benefits that favour the radial approach as the default vascular access site in ACS patients who undergo PCI [22]. This study showed that for the invasive management of ACS, when compared to femoral access, radial access significantly reduces the net adverse clinical events mainly through a decrease in.

major bleeding and all-cause death (Hazard ratio 0.83; 95% CI 0.74–0.96; p = 0.0092 and Hazard ratio 0.72; 95% CI 0.53–0.99; p = 0.045, respectively).

Finally, sustained optimal primary and secondary prevention therapy reduces thirty-day and one-year mortality in ACS patients. Dual antiplatelet therapy (DAPT) (i.e., a combination of aspirin and P2Y12 inhibitors such as clopidogrel, ticagrelor or prasugrel) is recommended for all STEMI patients. DAPT, oral beta-blockers and angiotensin-converting enzyme inhibitors decrease all-cause mortality after a STEMI [23]. High-intensity statins significantly reduce ischaemic stroke events in STEMI patients [24]. These agents prove most beneficial when initiated early in patients with no contra-indications.

This study seeks to determine the event rates of crude mortality, crude survival, re-hospitalisation, heart failure, angina, major bleeding, thromboembolism, arrhythmias and revascularisation at 30 days, three months, six months and one year after the index STEMI. We seek to compare these clinical outcomes between four primary STEMI sub-groups (i.e., those who receive fibrinolysis vs. no fibrinolysis vs. PCI vs. no PCI) in our local practice and compared with those reported in HIC and other regions of SSA. We also aim to evaluate the association between the peak baseline measurements of serum hs-cTnT, serum CRP, SUA and clinical outcomes within a year.

We hypothesise that the thirty-day and one-year crude mortality rates for STEMI patients observed in our setting would follow similar trends to those in HIC with no significant differences. We anticipate that the various STEMI management therapies provided in our practice would offer similar one-year all-cause mortality and morbidity benefits, irrespective of the revascularisation strategy used when comparing fibrinolysis vs. PPCI vs. fibrinolysis followed by PCI among groups. In our setting, we expect that advanced age, female gender, diabetes mellitus and heart failure on admission play critical roles in predicting death at 30 days and one year.

## Methods

### Study aims

This study aims to determine the clinical outcomes of an acute STEMI within one year of clinical presentation to the division of cardiology at the CMJAH. The study also evaluates the association between baseline serum cardiac biomarkers of myocardial infarction and all-cause mortality within a year of acute STEMI.

The primary objective is:

- To determine the all-cause mortality rate of an acute STEMI at 30 days, three and six months, and one year after the index presentation.

The secondary objectives include:

- To determine the rates of cardiac-related re-hospitalisation, heart failure, angina, major bleeding, thromboembolism, non-fatal arrhythmias (namely, atrial fibrillation, ventricular tachycardia, and complete heart block) and planned or unplanned repeated revascularisation at 30 days, three and six months and one year after the index STEMI at CMJAH.
- To assess the relationship between all-cause mortality and the peak serum hs-cTnT, CRP and SUA measured on admission.
- To describe the baseline clinical characteristics of STEMI patients in our setting.
- To compare the clinical outcomes between STEMI patients who received thrombolysis, PCI, a pharmaco-invasive strategy and those who underwent no reperfusion at 30 days, six and three months, and one year after the index STEMI.
- To determine the baseline clinical predictors of all-cause mortality at 30 days, three months, six months and one year after the initial STEMI.
- To describe the adjuvant medical care provided and medication compliance rates at 30 days, three months, six months and one year after the index discharge from the hospital.

### Study design and setting

The study is a single-centre, observational, prospective cohort of all consecutive individuals with an acute STEMI diagnosis presenting to the CMJAH. The study will take place in the division of cardiology at the CMJAH, a academic state-owned tertiary PCI-capable healthcare facility in Johannesburg, South Africa. Participants will be followed over one year, and clinical outcomes will be measured at 30 days, three months, six months and one year from the index date of hospital discharge. The first day after hospitalisation will be considered day one of the follow-up.

#### Characteristics of study participants

All consecutive patients presenting to CMJAH with an acute STEMI diagnosis will be invited to participate in the study as shown in the flow diagram provided in Appendix A.

Inclusion criteria include:

- Age ≥ 18 years.
- All confirmed acute STEMI diagnoses as per the fourth universal definition of myocardial infarction, i.e., myocardial injury evidenced by a rise and/or fall of serum cardiac troponin levels with at least one value above the 99th percentile of the upper reference limit, in the presence of the following, ischaemic symptoms and new ischaemic ECG changes, i.e., persistent ST-segment elevation of at least 1 mm measured from the J-point in at least two contiguous leads, or new pathological Q-waves or a new-onset left bundle branch block.
- All confirmed STEMI receiving the various STEMI management, i.e., thrombolysis, PCI, pharmaco-invasive approach, and no reperfusion.
- Able to give written informed consent.
- The participant or participant’s next of kin is reachable by cellphone.

Exclusion criteria include:

- ACS other than STEMI, i.e., NSTEMI and unstable angina.
- Unable to give consent.
- Unreachable by telephone call (participant or their next of kin).
- Incomplete medical records.

### Data collection

Research data will be collected from 01 May 2021 to 30 October 2022. The Primary Investigator (PI) will use the Research Electronic Data Capture (REDCap) software as the primary data collection tool. At baseline, the PI will capture demographic, clinical, calculated TIMI and GRACE scores, laboratory test results, ECG, echocardiographic, coronary angiographic, revascularisation and discharge medication data shown in Appendix B. The PI will create and access a reference list of the study participants’ hospital numbers and specific study numbers. The PI will retrieve these from the relevant progress notes, National Health Laboratory Service (NHLS) lab track, electronic medical records, and coronary angiogram report database of the division of cardiology at CMJAH.

The PI will telephonically contact all participants or their designated family members to monitor general well-being, medication compliance, outpatient cardiac clinic visits and cardiac-related re-admissions. When re-admitted to CMJAH, the PI will access the relevant hospital records to ascertain whether our study’s outcomes of interest occurred. Participants will be deemed compliant with medication if they say so during the telephonic interviews. The telephonic interviews will follow a closed-ended structured questionnaire, as depicted in Appendix C. Each participant will receive a telephonic interview call at 30 days, three months, six months and twelve months after the index hospital discharge date. All participants will be given the PI’s phone number and business card and encouraged to save the number on their cellphones when they sign informed consent forms so that doctors from other hospitals can contact the PI for cardiac-related referrals to CMJAH.

The PI will obtain the peak values of serum hs-cTnI, serum CRP and SUA from routine blood tests drawn at baseline for all participants. Automated messages generated through REDCap will be sent to remind both the PI and the participants of their scheduled outpatient clinic visits a week before the visits.

An experienced research sonographer will perform standardised echocardiograms during the outpatient clinic visits three months and one year after the index discharge. The two cardiologists who supervise the study will verify all echocardiograms. Appendix D shows the left ventricular parameters and valve analysis that will be recorded.

### Study sample size calculation

A minimum sample size of 355 participants in one year is required to achieve a study power of at least 80% for statistical significance. Using Cochrane’s formula, a minimum sample size of 182 participants is required for statistical significance with a 95% confidence interval and a 5% margin of error to assess the clinical outcomes at 30 days. Appendix E illustrates the formulae and sample size calculations used.

### Statistical analysis

Stata Corp. IC Version 16.1 will be used for all statistical analyses. Normally distributed continuous data will be presented as means with standard deviations (± SD). Continuously skewed data will be reported as medians with interquartile ranges (IQR). We will express categorical data as numbers and percentages. For intergroup comparisons, the student’s unpaired t-test and one-way analysis of variance (ANOVA) with Fisher’s test will be used for continuous variables and the chi-square test for categorical variables. Demographic and clinical profiles will be presented in frequency tables. Univariate and multivariate cox regression analyses will be used to determine the baseline clinical predictors of mortality. Survival curves generated by the Kaplan-Meier method will be used to determine the crude one-year survival among the different reperfusion groups and compared with log-rank tests. All variables will be expressed with their hazard ratio and 95% confidence intervals. For all tests, a p-value < 0.05 will be considered statistically significant.

## Discussion

This study will describe the demographic, risk factors and clinical profiles of individuals with STEMI in South Africa. In addition, the study will provide insights into the routine use of baseline serum uric acid as a potential low-cost prognostic biomarker of all-cause mortality in STEMI. Its findings may improve the risk stratification, prognostication, and therapeutic management of STEMI patients in our setting, particularly in sub-Saharan Africa, where access to emergency services and a cardiac catheterisation laboratory may be limited.

This current registry is significant because the South African ACCESS registry was conducted nearly 15 years ago in 29 private hospitals across South Africa predominantly treating Caucasians [6]. Although its results are still relevant, they have become outdated as STEMI treatment guidelines have changed, and management protocols evolved. Furthermore, compared to the South African ACCESS registry, which enrolled 615 participants, of which nearly 253 participants had an acute STEMI, this cohort aims to recruit at least 355 acute STEMI patients. Moreover, this research is conducted at a large state-owned tertiary facility predominantly treating black Africans, which could thus be more representative of the South African population than previous studies. In addition, it provides detailed insights into the different clinical outcomes of the various coronary reperfusion therapies of acute STEMI in our setting, as outlined in our data collection appendices. Finally, this research supplements the existing literature because it offers insights into the clinical utility of serum uric acid as a potential low-cost prognostic biomarker of all-cause mortality, particularly amongst Africans presenting with an acute STEMI diagnosis.

This study has several potential limitations. First, it is a single-centre study and thus prone to selection bias. Second, its observational nature may not allow us to control for unmeasured confounders that may contribute to the clinical outcomes observed. Third, although participants’ and participants’ next of kin’s cellphone numbers will be recorded, the prospective nature of the study makes it susceptible to loss to follow-up. Fourth, the follow-ups will be conducted through structured telephonic interviews, which may lead to patients omitting vital health data and, thus, under-reporting adverse cardiovascular events of interest to this study. Finally, the study site’s emergency department was not fully functional due to a fire incident that occurred prior to the start of the study, which may limit the number of participants presenting at our facility.

### Electronic supplementary material

Below is the link to the electronic supplementary material.

Additional File 1: APPENDIX A_Flow diagram showing the study population that will be recruited for the cohort.

Additional File 2: STEMI Demographics, Clinical and Angiographic Data.

Additional File 3: Periodic Telephonic Interview and Clinical Outcomes.

Additional File 4: Outpatient Echocardiogram.

Additional File 5: APPENDIX E.

## Data Availability

The datasets used and analysed during the current study are available from the corresponding author upon reasonable request.

## Abbreviations

ACS: Acute coronary syndrome
AMI: Acute myocardial infarction
CMJAH: Charlotte Maxeke Johannesburg Academic Hospital
DAPT: Dual antiplatelet therapy
ECG: Electrocardiogram
HIC: High-income countries
CRP: C-reactive protein
hs-cTn: High-sensitivity cardiac troponin
IHD: Ischaemic heart disease
LBBB: Left bundle branch block
LMIC: Low-to-middle-income countries
MI: Myocardial infarction
NSTEMI: Non-ST segment elevation myocardial infarction
PCI: Percutaneous coronary intervention
PI: Primary investigator
PPCI: Primary percutaneous coronary intervention
PTCA: Percutaneous transluminal coronary angioplasty
SSA: Sub-Saharan Africa
STEMI: ST-segment elevation myocardial infarction
SUA: Serum uric acid
