# Supplementary material for: ST-segment elevation myocardial infarction heart of Charlotte one-year (STEMI HOC-1) study: a prospective study protocol
Source: BMC Cardiovasc Disord. 2023 Aug 11;23:396. doi: 10.1186/s12872-023-03416-3 (PMC10422761; doi:10.1186/s12872-023-03416-3)
Supplement: Supplementary file 1 — Additional File 1: APPENDIX A_Flow diagram showing the study population that will be recruited for the cohort. [file 12872_2023_3416_MOESM1_ESM.docx]

APPENDIX A_ Flow diagram showing the study population that will be recruited for the cohort.

Patients presenting at CMJAH with an acute STEMI diagnosis

(Transfers and ED visits)

Thrombolysis (pre-hospital or in-hospital)

No thrombolysis

Coronary angiography?

Yes

No

PCI

No PCI
