# Supplementary material for: ST-segment elevation myocardial infarction heart of Charlotte one-year (STEMI HOC-1) study: a prospective study protocol
Source: BMC Cardiovasc Disord. 2023 Aug 11;23:396. doi: 10.1186/s12872-023-03416-3 (PMC10422761; doi:10.1186/s12872-023-03416-3)
Supplement: Supplementary file 2 — Additional File 2: STEMI Demographics, Clinical and Angiographic Data. [file 12872_2023_3416_MOESM2_ESM.docx]

Appendix B

*Management And One-Year Clinical Outcomes of ST-segment Elevation Myocardial Infarction*

*Page 1*

STEMI Demographics, Clinical and Angiographic Data

Record ID

**DEMOGRAPHICS**

Hospital number

Date of admission

First name

Last name

Gender
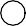
 Male
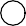
 Female

Date of birth

Age at time of hospital admission

Self-identified race Black

Asian Coloured Indian White Other


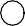

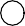

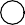

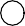

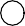

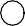


Cellphone number of study participant

Cellphone number of next of kin

Weight (Kg)

Height (meters)

Body Mass Index

Waist circumference (cm)

**COMORBIDITIES**

Comorbidities

Diabetes (IDDM/NIDDM) Hypertension Dyslipidaemia

Obesity

Chronic Kidney disease Peripheral vascular disease

Family history of heart disease (myocardial infarction) Physical inactivity

Menopause/ Post-menopause (if female) Previous myocardial infarction

History of stroke/ transient ischaemic attack Human immunodeficiency virus

None of the above

other (e.g. gout, malignancy etc.)

If other comorbidities, please specify (e.g. gout)

If diabetic insulin-dependent diabetes mellitus (IDDM)

non-insulin-dependent diabetes mellitus (NIDDM)


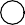

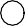


HbA1c at admission (%)

CD4+ count

Viral load
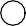
 Supressed
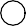
 Not suppressed

Cigarette smoking Active smoker
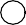
 Ex-smoker

Never smoked


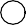

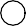


Please specify the number of cigarette smoking pack-years (years)

Psychosocial stressors (i.e. diagnosed major depressive disorder and other mood disorders, anxiety disorders, substance use disorders, psychotic disorders and other social stressors as reported by

the patient)


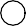
 Yes
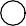
 No
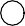
 Unknown

Prior PCI
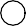
 Yes
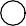
 No

Previous PCI date (i.e. numeric month and year)

Prior coronary artery bypass graft (CABG)
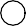
 Yes
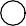
 No

Previous coronary artery bypass graft (CABG) date (i.e. numeric month and year)


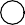

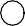

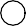

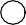


| **CLINICAL PRESENTATION ON ADMISSION** |  | |
| --- | --- | --- |
| Presence of chest pain? | Yes No |  |
| The severity of chest pain on a scale of 1-10 (based on patient history) |  |  |
|  |  |  |
| Angina | CCS I 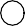 CCS II CCS IV | 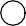 CCS III |
| Date and time of onset of peak (maximal) angina |  |  |
|  |  |  |
| Atypical symptoms of an acute MI | Dyspnoea Fatigue Palpitations Sweating Nausea Vomiting Syncope  None of the above |  |
| Systolic blood pressure at admission (mmHg) |  |  |
|  |  |  |
| Diastolic blood pressure at admission (mmHg) |  |  |
|  |  |  |
| Heart failure at admission (i.e. presence of clinical signs of heart failure using the Killip Classification) | 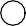 Yes 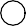 No |  |
| Cardiogenic shock at admission (i.e. a systolic blood pressure < 90 mmHg) | 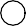 Yes 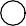 No |  |
| NYHA Class | 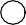 I 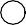 II 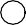 III | IV |
| Cardiac arrest at admission (i.e. required CPR, or direct current cardioversion or defibrillation) | 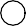 Yes 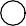 No |  |

Killip classification (I, II, III, IV) Class I: no clinical signs of heart failure

Class II: pulmonary rales or crackles, S3 gallop, elevated jugular venous pressure


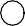

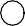


Class III: overt acute pulmonary oedema Class IV: cardiogenic shock, systolic blood


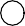

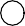


pressure < 90mmHg or evidence of peripheral vasoconstriction (oliguria, cyanosis, sweating)

Global Registry of Acute Coronary Events (GRACE 2.0) risk of in-hospital mortality (%) calculated by QxMD

Thrombolysis in myocardial infarction (TIMI) Score for STEMI (points) calculated by MDCalc

Thrombolysis in myocardial infarction (TIMI) mortality

risk assessment at 30 days after discharge (%) calculated by MDCalc

Global Registry of Acute Coronary Events (GRACE)

mortality risk assessment score at 6 months (points) calculated by MDCalc

Global Registry of Acute Coronary Events (GRACE)

mortality risk assessment score at 6 months (%) calculated by MDCalc

Global Registry of Acute Coronary Events (GRACE 2.0)

mortality risk assessment score at 12 months (%) calculated by QxMD

**ECG diagnostic features**

Date and Time of STEMI diagnosis on ECG (i.e. 'time

zero' to evidence-based therapy)

Time from onset of peak angina to ECG diagnosis (in days)

cardiac rhythm (based on ECG)

sinus

Atrial fibrillation Atrial flutter

Supraventricular tachycardia (SVT) Ventricular tachycardia (VT)

First-degree AV Block

Second-degree AV block (Mobitz I) Second-degree AV block (Mobitz II) Third-degree AV block

Other abnormal cardiac rhythms (e.g. fascicular block)

If other abnormal cardiac rhythms, please specify (e.g. left/right fascicular block)

Heart rate at admission (beats per minute)

12 lead ECG leads showing ST-segment elevation

V1 V2 V3 V4 V5 V6

lead I lead II lead III aVL avR avF

None of the above (i.e. deep Q-waves only / late-presenting STEMI)

STEMI location based on interpretation of 12 lead ECG

Anterior wall (V3, V4)

Antero-lateral wall (V3-V6, I, avL) Septal (V1-V2)

Lateral wall (I, avL, V5, V6) Inferior wall (II, III, avF)

Inferolateral wall (I, II, III, avF, V5-V6)

Posterior wall (V1-V4 showing reciprocal ST depression, tall, broad R waves with R/S ratio > 1, upright T waves)

Right ventricular wall (V1, V4R, III)

Pathological Q-waves
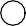
 Yes
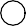
 No

ECG leads showing pathological Q-waves

V1 V2 V3 V4 V5 V6

lead I lead II lead III avL avR avF

ECG interpretation of pathological Q-waves

Anterior wall (V3, V4) Lateral wall (I, avL, V5, V6) Inferior wall (II, III, avF)

Posterior wall (posterior leads V7,V8,V9 or reciprocal ST-depression V3,V4,V5)

Presence of a bundle branch block Left bundle branch block Right bundle branch block None


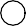

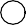

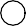


Please upload the first ECG tracing showing a STEMI diagnosis from the referral site or from CMJAH on admission

**BLOOD BIOCHEMICAL RESULTS ON ADMISSION**

Type of serum cardiac troponin measured

High-sensitivity cardiac troponin T (hs-cTnT) High-sensitivity cardiac troponin I (hs-cTnI) Troponin T

Troponin I

Serum high-sensitivity cardiac troponin T peak value (ng/L)

Serum high-sensitivity cardiac troponin I peak value (ng/L)

Serum cardiac troponin T peak value (ng/mL)

conversion of troponin T value to high-sensitivity troponin equivalent (ng/L)

| Serum cardiac troponin I peak value (ng/mL) |  | | |
| --- | --- | --- | --- |
|  |  |  |  |
| conversion of troponin I value to high-sensitivity troponin equivalent (ng/L) |  |  |  |
|  |  |  |  |
| At least one cardiac troponin value above the 99th percentile upper reference limit | 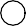 Yes | No |  |
| Rise and subsequent fall of cardiac troponins | 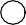 Yes | No |  |
| CK-MB peak value (Ug/L) |  |  |  |
|  |  |  |  |
| Baseline C-reactive protein (CRP) (mg/L) |  |  |  |
|  |  |  |  |
| Baseline serum uric acid (mmol/L) |  |  |  |
|  |  |  |  |
| Baseline serum sodium (Na+) (mmol/L) |  |  |  |
|  |  |  |  |
| Baseline serum potassium (K+) (mmol/L) |  |  |  |
|  |  |  |  |
| Baseline serum urea (mmol/L) |  |  |  |
|  |  |  |  |
| Baseline serum creatinine (Umol/L) |  |  |  |
|  |  |  |  |
| Baseline creatinine clearance (ml/min) using the Cockcroft-Gault formula calculated using MDCalc |  |  |  |
|  |  |  |  |
| Total cholesterol (mmol/L) |  |  |  |
|  |  |  |  |
| Triglycerides (mmol/L) |  |  |  |
|  |  |  |  |
| HDL cholesterol (mmol/L) |  |  |  |
|  |  |  |  |
| LDL cholesterol (mmol/L) |  |  |  |
|  |  |  |  |
| White cell count ( x 10^9/ L) |  |  |  |
|  |  |  |  |
| Baseline serum haemoglobin (g/dL) |  |  |  |

**ECHOCARDIOGRAM FINDINGS ON ADMISSION**

Left Ventricular Internal Dimension in diastole (LVIDd) in millimetres (mm)

((in millimeters))

Left Ventricular Internal Dimension in systole (LVIDs) in millimetres (mm)

((in millimeters))

Echo LVEF (%) at admission 0 50 100

*(Place a mark on the scale above)*

Left atrial size in millimetres (mm)

((in millimeters))

LV regional wall motion abnormality (RWMA)

Anterior Posterior Inferior Lateral Septal Global Apical

None of the above ((on echocardiography))

Valve screen pathology

Aortic stenosis Aortic regurgitation Mitral stenosis Mitral regurgitation Tricuspid stenosis

Tricuspid regurgitation Pulmonary stenosis Pulmonary regurgitation None of the above

((on echocardiography))

LV Clot Yes


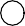

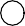

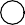


No Unknown

((on echocardiography))

LA Clot Yes

No Unknown


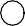

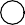

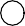


((on echocardiography))

| **CARDIAC CATHETERISATION** |  | |
| --- | --- | --- |
| Was a diagnostic coronary angiogram (DCA) done? | 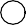 Yes | No |
| Date and time of index coronary angiogram |  |  |
|  |  |  |
| Time duration from qualifying ECG (STEMI diagnosis i.e. 'time zero') to diagnostic coronary angiogram |  |  |
| (DCA) (in days) |  |  |
| Time duration from symptom onset to diagnostic coronary angiogram (DCA) (in days) |  |  |
|  |  |  |
| Was an intervention (i.e. PCI) performed? | 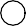 Yes | No |
| Date and time of PCI |  |  |
|  |  |  |
| Duration between time from qualifying ECG (STEMI diagnosis i.e. 'time zero') to PCI (in days) |  |  |
|  |  |  |
| Duration between symptom onset to needle/balloon time (in days) |  |  |
|  |  |  |
| Fibrinolysis administered? (e.g. alteplase, streptokinase) | 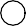 Yes | No |

If yes, Fibrinolysis administered before admission to CMJAH

Fibrinolysis administered during current admission to CMJAH


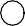

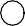


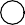
 unknown whether fibrinolysis was given before or during current CMJAH admission

Type of fibrinolytic agent administered Alteplase (i.e. actilyse/ activase) Streptokinase

Metalyse (i.e. tenecteplase) Unknown


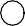

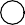

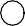

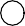


Date and time of fibrinolysis

Duration from symptom onset to fibrinolysis (in days)

STEMI definitive management subgroup Thrombolysis only

Thrombolysis and diagnostic coronary angiogram (DCA) without PCI


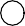

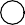


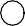
 Diagnostic coronary angiogram (DCA) with PCI (i.e. no prior thrombolysis)


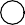
 Thrombolysis and diagnostic coronary angiogram (DCA) with PCI


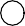
 Diagnostic coronary angiogram (DCA) only (i.e. no thrombolysis and no PCI)


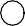
 Optimal medical therapy (OMT) and supportive management only (i.e. no DCA and no reperfusion by thrombolysis and PCI)

Revascularisation strategy Thrombolysis (with/ without DCA)


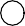

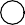


Primary PCI (i.e. patient with no prior thrombolysis taken directly to the cath lab for PCI)


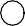
 Pharmacoinvasive strategy (i.e. failed thrombolysis followed by DCA + rescue PCI)


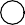
 No reperfusion (i.e. patient received OMT or DCA only)


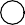

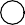

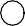


Coronary reperfusion group Thrombolysis (with and without DCA) PCI (with and without prior thrombolysis) No reperfusion

Culprit coronary artery (i.e. vessel with an acute thrombotic total or subtotal occlusion causing myocardial infarction) detected on coronary angiogram

Left main stem LAD

Diagonal

Left circumflex Obtuse marginal RCA

Posterior descending Acute marginal Ramus

None of the above

Diseased vessels on coronary angiography

Left main stem LAD

Diagonal

Left circumflex Obtuse marginal RCA

Posterior descending Acute marginal Ramus

None of the above

Is the left main stem lesion in the proximal, middle or distal segment?

Proximal Distal

Middle

The extent of left main stem stenosis on coronary angiography (%)

< 50% 50% - 70%

> 70% ≥ 90% (sub-total occlusion) total occlusion


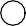

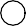

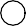

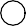

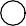


Was the instantaneous wave-free ratio (iFR) measured?
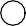
 Yes
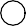
 No

iFR value in Left main stem

Is the LAD lesion in the proximal, middle or distal segment?

Proximal Distal

Middle

The extent of the LAD stenosis on coronary angiography (%)

< 50% 50% - 70%

> 70% ≥ 90% (sub-total occlusion) total occlusion


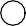

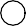

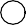

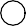

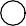


Was the iFR measured?
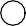
 Yes
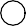
 No

iFR value in LAD lesion

Is the RCA lesion in the proximal, middle or distal segment?

Proximal Distal

Middle

The extent of RCA stenosis on coronary angiography (%) < 50% 50% - 70%


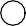

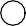

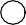

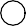

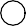


> 70% ≥ 90% (sub-total occlusion) total occlusion

Was the iFR measured?
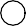
 Yes
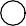
 No

iFR value in RCA

Is the left circumflex lesion in the proximal, middle or distal segment?

Proximal Distal

Middle

The extent of left circumflex stenosis on coronary angiography (%)

< 50% 50% - 70%

> 70% ≥ 90% (sub-total occlusion) total occlusion


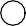

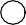


Was the iFR measured? Yes No

iFR value in left circumflex

Is the OM lesion in the proximal, middle or distal segment?

Proximal Distal

Middle

The extent of the obtuse marginal (OM) stenosis on coronary angiography (%)

< 50% 50% - 70%

> 70% ≥ 90% (sub-total occlusion) total occlusion

Was the iFR measured? Yes No

iFR value in OM

Is the diagonal lesion in the proximal, middle or distal segment?

Proximal Distal

Middle

The extent of the diagonal stenosis on coronary angiography (%)

< 50% 50% - 70%

> 70% ≥ 90% (sub-total occlusion) total occlusion

Was the iFR measured? Yes No

iFR value in diagonal

Is the PDA lesion in the proximal, middle or distal segment?

Proximal Distal

Middle

The extent of the PDA stenosis on coronary angiography (%)

< 50% 50% - 70%

> 70% ≥ 90% (sub-total occlusion) total occlusion

Was the iFR measured? Yes No

iFR value in PDA

Is the acute marginal (AM) lesion in the proximal, middle or distal segment?

Proximal Distal

Middle

The extent of the acute marginal (AM) stenosis on coronary angiography (%)

< 50% 50% - 70%

> 70% ≥ 90% (sub-total occlusion) total occlusion

Was the iFR measured? Yes No

iFR value in the acute marginal

Is the ramus lesion in the proximal, middle or distal segment?

Proximal Distal

Middle

The extent of the ramus stenosis on coronary angiography (%)

< 50% 50% - 70%

> 70% ≥ 90% (sub-total occlusion) total occlusion

Was the iFR measured? Yes No

iFR value in the ramus

Non-diseased vessels on coronary angiography

Left main stem LAD

Diagonal

Left circumflex Obtuse marginal RCA

Posterior descending Acute marginal

All of the above

number of lesions Single vessel disease

Double vessel disease Triple vessel disease

Multivessel disease with >3 vessels involved None of the above

PCI modality

Drug eluting stent (DES) Drug eluting balloon (DEB) Bare metal stent (BMS)

Percutaneous trans-luminal coronary angioplasty (PTCA), i.e. balloon angioplasty

Aspiration thrombectomy (thrombus aspiration)

Coronary vessels reperfused

left main coronary artery LAD

RCA PDA

Left circumflex Obtuse marginal Diagonal

Ramus

None of the above

Thrombolysis in myocardial infarction (TIMI) grade TIMI flow grade 0 (no antegrade flow, artery is flow in left main coronary artery on angiogram after completely occluded)

reperfusion TIMI flow grade 1 a (dye minimally leaks past the area of obstruction)

TIMI flow grade 1 b (dye leaks past the area of obstruction but fails to opacify the entire coronary bed)

TIMI flow grade 2 a (slow flow, dye markedly delayed in opacifying distal vasculature)

TIMI flow grade 2 b (fast flow, dye minimally delayed in opacifying distal vasculature)

TIMI flow grade 3 (normal flow in the artery)

Thrombolysis in myocardial infarction (TIMI) grade TIMI flow grade 0 (no antegrade flow, artery is flow of LAD on angiogram after reperfusion completely occluded)

TIMI flow grade 1 a (dye minimally leaks past the area of obstruction)

TIMI flow grade 1 b (dye leaks past the area of obstruction but fails to opacify the entire coronary bed)

TIMI flow grade 2 a (slow flow, dye markedly delayed in opacifying distal vasculature)

TIMI flow grade 2 b (fast flow, dye minimally delayed in opacifying distal vasculature)

TIMI flow grade 3 (normal flow in the artery)

Thrombolysis in myocardial infarction (TIMI) grade TIMI flow grade 0 (no antegrade flow, artery is flow of RCA on coronary angiogram after reperfusion completely occluded)

TIMI flow grade 1 a (dye minimally leaks past the area of obstruction)

TIMI flow grade 1 b (dye leaks past the area of obstruction but fails to opacify the entire coronary bed)

TIMI flow grade 2 a (slow flow, dye markedly delayed in opacifying distal vasculature)

TIMI flow grade 2 b (fast flow, dye minimally delayed in opacifying distal vasculature)

TIMI flow grade 3 (normal flow in the artery)

Thrombolysis in myocardial infarction (TIMI) grade TIMI flow grade 0 (no antegrade flow, artery is flow of the left circumflex artery on coronary completely occluded)

angiogram after reperfusion TIMI flow grade 1 a (dye minimally leaks past the area of obstruction)

TIMI flow grade 1 b (dye leaks past the area of obstruction but fails to opacify the entire coronary bed)

TIMI flow grade 2 a (slow flow, dye markedly delayed in opacifying distal vasculature)

TIMI flow grade 2 b (fast flow, dye minimally delayed in opacifying distal vasculature)

TIMI flow grade 3 (normal flow in the artery)

Thrombolysis in myocardial infarction (TIMI) grade TIMI flow grade 0 (no antegrade flow, artery is flow of PDA on angiogram after reperfusion completely occluded)

TIMI flow grade 1 a (dye minimally leaks past the area of obstruction)

TIMI flow grade 1 b (dye leaks past the area of obstruction but fails to opacify the entire coronary bed)

TIMI flow grade 2 a (slow flow, dye markedly delayed in opacifying distal vasculature)

TIMI flow grade 2 b (fast flow, dye minimally delayed in opacifying distal vasculature)

TIMI flow grade 3 (normal flow in the artery)

Thrombolysis in myocardial infarction (TIMI) grade TIMI flow grade 0 (no antegrade flow, artery is flow of the obtuse marginal artery on angiogram after completely occluded)

reperfusion TIMI flow grade 1 a (dye minimally leaks past the area of obstruction)

TIMI flow grade 1 b (dye leaks past the area of obstruction but fails to opacify the entire coronary bed)

TIMI flow grade 2 a (slow flow, dye markedly delayed in opacifying distal vasculature)

TIMI flow grade 2 b (fast flow, dye minimally delayed in opacifying distal vasculature)

TIMI flow grade 3 (normal flow in the artery)

Thrombolysis in myocardial infarction (TIMI) grade TIMI flow grade 0 (no antegrade flow, artery is flow of diagonal artery on angiogram after reperfusion completely occluded)

TIMI flow grade 1 a (dye minimally leaks past the area of obstruction)

TIMI flow grade 1 b (dye leaks past the area of obstruction but fails to opacify the entire coronary bed)

TIMI flow grade 2 a (slow flow, dye markedly delayed in opacifying distal vasculature)

TIMI flow grade 2 b (fast flow, dye minimally delayed in opacifying distal vasculature)

TIMI flow grade 3 (normal flow in the artery)

Thrombolysis in myocardial infarction (TIMI) grade TIMI flow grade 0 (no antegrade flow, artery is flow of ramus artery on angiogram after reperfusion completely occluded)

TIMI flow grade 1 a (dye minimally leaks past the area of obstruction)

TIMI flow grade 1 b (dye leaks past the area of obstruction but fails to opacify the entire coronary bed)

TIMI flow grade 2 a (slow flow, dye markedly delayed in opacifying distal vasculature)

TIMI flow grade 2 b (fast flow, dye minimally delayed in opacifying distal vasculature)

TIMI flow grade 3 (normal flow in the artery)

Coronary catheterisation vascular access site

Right radial artery Left radial artery Right femoral artery Left femoral artery Other

Failed RRA converted to RFA Failed RRA converted to LFA

If another vascular access site, please specify

Coronary angiogram start time

Coronary angiogram end time

Total coronary angiogram procedure duration (in minutes)

**COMPLICATIONS**

In-hospital complications

Cardiac

Acute stent thrombosis

Repeated diagnostic coronary angiogram (e.g. for staged PCI or stent relook/ re-do) Peripheral thromboembolism (e.g. deep venous thrombosis (DVT), pulmonary embolism (PE)) Neurological (stroke/transient ischaemic attack)

Major bleeding (defined as intracranial hemorrhage, Hb decrease > 2g/dL, required hospitalisation and/ or a blood transfusion)

Acute kidney injury/ acute renal failure Death

Other

None of the above

Other complications (please specify)

Death during first hospitalisation? Yes No

Unknown

If yes, date of death

Cause of death

Neurological (stroke)

Cardiac (MI, heart failure, VF/pulseless VT, cardiac arrest)

Pulmonary (pulmonary embolism, respiratory infection)

COVID-19

Haemodynamic instability unknown

Other

Other causes of death (please specify)

Time duration between ECG STEMI diagnosis ('time zero) and death (in days)

**DISCHARGE INFORMATION**

Date of discharge

Duration of hospitalisation

Medication prescribed at discharge

Dual antiplatelet therapy (DAPT i.e. aspirin and clopidogrel/plavix)

Aspirin only

P2Y12 inhibitors (e.g. clopidogrel/plavix) only Heparin (low molecular weight heparin (e.g. enoxaparin), unfractionated heparin)

Beta-blocker (e.g. atenolol, carvedilol) ACE-I (e.g. enalapril)

ARB (e.g. losartan, valsartan)

Statin (e.g. atorvastatin, simvastatin) Mineralocorticoid receptor antagonist (MRA) (e.g. aldactone, spirinolatone)

None of the above Other

Other medications prescribed

Referral for coronary artery bypass graft (CABG) Yes No Unknown

If yes, date of CABG (if known)

**Health Records**

Please upload the coronary angiogram report

Please upload other relevant health records (e.g. cardiology ticksheets, ECGs, flow charts, progress notes, referral letters and discharge summary etc.)

Please upload the Participant Consent Sheet
