# Supplementary material for: ST-segment elevation myocardial infarction heart of Charlotte one-year (STEMI HOC-1) study: a prospective study protocol
Source: BMC Cardiovasc Disord. 2023 Aug 11;23:396. doi: 10.1186/s12872-023-03416-3 (PMC10422761; doi:10.1186/s12872-023-03416-3)
Supplement: Supplementary file 3 — Additional File 3: Periodic Telephonic Interview and Clinical Outcomes. [file 12872_2023_3416_MOESM3_ESM.docx]

Appendix C

*Management And One-Year Clinical Outcomes of ST-segment Elevation Myocardial Infarction*

*Page 1*

Periodic Telephonic Interview and Clinical Outcomes

Record ID

**Telephonic Interview**

Date of data collection

Data obtained from study participant

study participant's next of kin (spouse/child/parent/sibling)


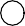

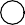


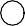
 primary investigator

Date of index hospital discharge

Time after index hospital discharge date (in days)

1. Has the participant visited the cardiac clinic at CMJAH since discharge?


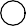
 Yes
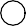
 No

If yes, please indicate date of visit

If yes, please indicate reason for visit

If yes, was an echo done at this visit?
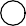
 Yes
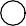
 No

Echo LVEF (%) 0 50 100

*(Place a mark on the scale above)*

1. Has the participant been re-admitted to the Division of Cardiology at CMJAH?


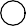
 Yes
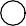
 No

If yes, please indicate date of re-admission

If yes, please indicate reason for re-admission

((from participant))

Reason for re-admission (from the patient medical records)

Subsequent myocardial infarction (STEMI or NSTEMI) Acute stent thrombosis

Elective admission for a diagnostic coronary angiogram (DCA)

Elective admission for PCI or staged PCI Coronary artery bypass graft surgery (CABG) Non-cardiac cause

Other cardiac-related indications for admission (e.g. pacemaker ICD box change etc.)

((from hospital records accessed by the PI))

If another cardiac-related reason for re-admission, please specify

((from hospital records accessed by the PI))

If not re-admitted to CMJAH, has the participant been re-admitted to another hospital for a cardiac-related cause?


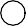
 Yes
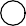
 No

If re-admitted to another hospital, please provide the name of the hospital

((from participant))

If re-admitted to another hospital, please provide the date of admission to the other hospital facility

((from participant))

If re-admitted to another hospital, please indicate the reason for admission to the other hospital

facility ((from participant))

1. Is the participant generally well and alive?
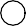
 yes
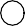
 No
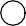
 Unknown

If alive, has the participant experienced persistent chest pain?


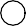
 Yes
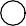
 No ((from participant))

If not alive, date of death (from next of kin or hospital records)

Time from index hospital discharge to death (in days)

Date of first STEMI ECG diagnosis

Time from first ECG STEMI diagnosis to death (in days)

Place of death Home

CMJAH


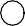

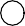

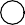

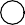


Other medical centre Unknown

If death occurred at another medical centre, please specify the location

1. Is the participant receiving medical therapy?
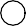
 Yes
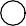
 No
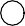
 Unknown

If yes, is the participant adherent to medication?
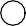
 Yes
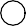
 No

If non-adherent, please indicate discontinued

Aspirin

DAPT

P2Y12 inhibitors

medication

(e.g. clopidogrel) Statin ACE-I

Beta-blocker ARB

Heparin (low molecular weight heparin e.g. enoxaparin, unfractionated heparin)

MRA (e.g. aldactone, spironolactone)

If non-adherent, please indicate reason

Medication cost

Medication adverse events/ side-effects Limited transport/access to local pharmacy or clinic

Personal choice

Discontinuation indicated by primary physician Other

If other, please specify e.g. course completed

**ADVERSE CLINICAL OUTCOMES**

Has any of the following complications occurred during the re-admission within the past month(s)?

Angina

Repeated coronary angiography Revascularisation

Acute Heart Failure Cardiogenic shock Major bleeding

Stroke/ TIA/ Thromboembolism Arrhythmia

None of the above

((from hospital records accessed by the PI))

Please specify if the repeated diagnostic coronary Planned (i.e. elective)


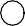

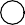

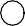


angiogram is: Unplanned (i.e. urgent)

Unknown whether planned or unplanned ((from hospital records accessed by the PI))

Please specify if the revascularisation is: Planned (i.e. elective) Unplanned (i.e. urgent)

Unknown whether planned or unplanned ((from hospital records accessed by the PI))


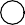

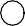

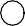


Please specify the arrhythmia

Atrial fibrillation

Ventricular

tachycardia Complete heart block

((from hospital records accessed by the PI))
