# Supplementary material for: ST-segment elevation myocardial infarction heart of Charlotte one-year (STEMI HOC-1) study: a prospective study protocol
Source: BMC Cardiovasc Disord. 2023 Aug 11;23:396. doi: 10.1186/s12872-023-03416-3 (PMC10422761; doi:10.1186/s12872-023-03416-3)
Supplement: Supplementary file 4 — Additional File 4: Outpatient Echocardiogram. [file 12872_2023_3416_MOESM4_ESM.docx]

Appendix D

*Management And One-Year Clinical Outcomes of ST-segment Elevation Myocardial Infarction*

*Page 1*

Outpatient Echocardiogram

Record ID

**Outpatient Echocardiogram**

Date of follow-up echocardiogram

Days from the index date of hospital discharge to follow-up echo (days)

((days))

Age at time of follow-up echo

Gender Male

Female


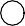

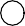


**Left Ventricular Function**

LVEF (%) at baseline echo

LVEF (%) on follow-up echo

Fractional Shortening (FS)

((%))

Stroke volume (SV) (ml)

((ml))

**Left Ventricular Regional Wall Function**

Normal Hypokinetic Dyskinetic Akinetic Bounce

Basal anterior Basal anteroseptal Basal inferior Basal inferolateral Basal anterolateral Basal inferoseptal Mid anterior

Mid anteroseptal Mid inferoseptal

Mid inferior

Mid inferolateral Mid anterolateral Apical anterior Apical septal Apical inferior Apical lateral Apex

Global hypokinesia Yes

No


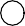

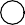


Interventricular Septum Thickness in diastole (IVSd) (mm)

((mm))

Left Ventricular Internal Dimension at end-systole (LVIDs) (mm)

((mm))

Left Ventricular Internal Dimension at end-diastole (LVIDd) (mm)

((mm))

Left ventricular end-systolic volume (LVES Vol) (ml)

((ml))

Left ventricular end-diastolic volume (LVED Vol) (ml)

((ml))

Left ventricular posterior wall thickness at end-diastole (LVPWd) (mm)

((mm))

Left Ventricular Mass Index (g/m2)

((g/m2))

Left Ventricular Outflow Tract Velocity- Time Integral (LVOT VTI) in centimetres per second (cm/s)

((cm/s))

Left atrium- Diameter (mm)

((mm))

Left atrium - Area (cm2)

((cm2))

Left atrium- Volume

((ml))

Left atrium- Index (ml/m2)

((ml/m2))

Mitral valve annulus (e)- Septal

((cm/s))

Mitral valve annulus (e)- Lateral

((cm/s))

Mitral valve annulus (e)- Average

((cm/s))

E/e' Ratio

E/A Ratio

LA Clot Yes

No


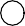

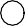


LV Clot Yes

No


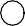

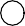


**Mitral Valve**

Mitral valve morphology Normal

Abnormal


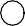

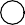


Mitral regurgitation (MR)- Severity Nil Trivial Mild

Moderate Severe


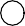

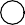

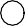

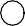

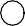


MR Aetiology Functional

Organic


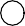

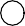

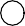


None of the above

Mitral stenosis (MS)- Severity Nil

Trivial Mild Moderate Severe


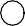

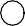

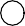

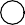

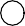


**Aortic Valve**

Aortic Valve Morphology Normal


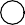

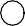


Abnormal

Aortic regurgitation (AR)- Severity Nil Trivial Mild

Moderate Severe


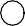

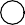

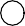

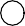

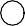


AR Aetiology Functional

Organic


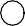

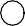

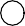


None of the above

Aortic stenosis (AS)- Severity Nil

Trivial Mild Moderate Severe


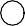

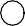

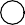

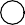

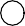


**Right Ventricular Function**

RV Dimensions- Basal RV Dimension (RVD1)

((mm))

RV Dimensions- Mid RV Dimensions (RVD2)

((mm))

RV Area- Diastolic

RV Area- Systolic

Tricuspid Valve Morphology Normal

Abnormal


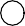

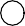


RV Function- Functional Area Change

((%))

RV Function- TAPSE

((mm))

**Right Atrial Function**

RA Volume

((ml))

Tricuspid Regurgitation- Severity Nil

Trivial Mild Moderate Severe


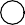

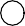

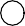

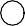

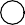


Peak velocity

((m/sec))

SPAP

((mmHg))

Inferior Vena Cava- Diameter

((cm))

Inferior Vena Cava- Reactive

((%))

Pericardial Effusion Nil

Mild Moderate Severe


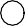

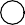

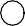

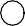


Type of pericardial effusion Fibrinous

Calcification


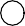

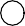

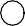


None of the above

**2D-STE Measurements**

Global longitudinal strain

((%))
