# Supplementary material for: ST-segment elevation myocardial infarction heart of Charlotte one-year (STEMI HOC-1) study: a prospective study protocol
Source: BMC Cardiovasc Disord. 2023 Aug 11;23:396. doi: 10.1186/s12872-023-03416-3 (PMC10422761; doi:10.1186/s12872-023-03416-3)
Supplement: Supplementary file 5 — Additional File 5: APPENDIX E. [file 12872_2023_3416_MOESM5_ESM.docx]

APPENDIX E

The formula used for sample size calculation is based on log-rank test which we will use for comparing survival between groups.

Below are the results for the sample size for assumptions made on these possible relationships:

1. In one year about 10% mortality in the PCI group – (which are at high risk of death)
2. Minimum 80% study power
3. Thrombolytic therapy 50% protective (used as default- smaller effects need much bigger samples)
4. Sample size ratio of 1.5 (less will get outcome from thrombolytic group with the same fixed period of 1 year follow-up as with PCI hence more patients needed in that group to have adequate events for comparison)
5. The estimated annual population size of STEMI patients in a pre-COVID-19 year at CMJAH is 1100 patients
6. The STEMI survival rate is 93.6 % after reperfusion by thrombolysis and 91.8% for PPCI, with no statistically significant difference

Power logrank 0.90, power (0.8) hratio(0.5) nratio(1.5)

Estimated sample sizes for two-sample comparison of survivor functions Log-rank test, Freedman method

H0: HR = 1 versus Ha: HR ! = 1

Study parameters: alpha = 0.0500

power = 0.8000

delta = 0.5000 (hazard ratio) hratio = 0.5000

N2/N1 = 1.5000

Censoring:

s1 = 0.9000

s2 = 0.9487

Pr_E = 0.0708

Estimated number of events and sample sizes:

| E = | 65 |
| --- | --- |
| N = | 907 |
| N1= | 363 |
| N2 = | 544 |
| N2/N1 = | 1.4986 |

We then used the finite population correction factor to adjust the calculated sample size based on these further assumptions:

1. Estimated annual STEMI population size presenting to CMJAH in a COVID-19 year 2020 = 650 STEMI patients

2021 = 580 STEMI patients (compounded effect of fire incident at CMJAH)

1. Estimated proportion that receives thrombolysis = 37 % (*figure from Steve Biko Hospital STEMI study*)

Sample size determination using the Finite Population Correction Factor in the formula below:

*n* = 𝑛0𝑁

𝑛0+ (𝑁 − 1)

Where n = sample size; N = STEMI population size in a COVID-19 year = 580 patients Total n = 355

N1= 143 (PCI group)

N2= 212 (Thrombolysis group)

For 30-day outcomes data, the following assumptions were made:

1. In Ethiopia, Fanta et al. included 111 STEMI patients and reported a 30-day all-cause mortality rate of 31.5% in this population group [1].
2. Our study will recruit a minimum study population of 400 patients in one year.
3. Using a 5% margin of error and 95% confidence interval

Using Cochran equation: n = $\frac{z^{2}pq}{e^{2}}$ , where n is the sample size, z is the standard error found in statistical tables which contain the area under the normal curve, i.e., z = 1.96 for 95% confidence interval, p is the population proportion, q = 1-p, and e is the margin of error.

| Confidence Level: |  |  |
| --- | --- | --- |
| Margin of Error: |  |  |
| Expected frequency: |  |  |
| Population Size: |  |  |
| \| Design effect: \|  \| \| --- \| --- \| \| Clusters: \| \|  \|    | |  |

Sample size: **182**

This means 182 or more measurements/surveys are needed to have a confidence level of 95% that the real value is within ±5% of the measured/surveyed value.

1. 1. Fanta K, Daba FB, Asefa ET, Melaku T, Chelkeba L, Fekadu G, et al. Management and 30-Day Mortality of Acute Coronary Syndrome in a Resource-Limited Setting: Insight From Ethiopia. A Prospective Cohort Study. Front Cardiovasc Med. 2021;8:707700.
